# CRISPR/Cas9-Mediated Editing of *BmEcKL1* Gene Sequence Affected Silk Gland Development of Silkworms (*Bombyx mori*)

**DOI:** 10.3390/ijms25031907

**Published:** 2024-02-05

**Authors:** Shimin Li, Junjie Lao, Yue Sun, Xiaoting Hua, Ping Lin, Feng Wang, Guanwang Shen, Ping Zhao, Qingyou Xia

**Affiliations:** 1 Integrative Science Center of Germplasm Creation in Western China (CHONGQING) Science City, Biological Science Research Center, Southwest University, Chongqing 400716, China; lishimin0203@163.com (S.L.); laojunjie2020@163.com (J.L.); huaxiaotingswu@126.com (X.H.); linpingswu@swu.edu.cn (P.L.); wangf1986@swu.edu.cn (F.W.); gwshen@swu.edu.cn (G.S.); zhaop@swu.edu.cn (P.Z.); 2 State Key Laboratory of Silkworm Genome Biology, Biological Science Research Center, Southwest University, Chongqing 400716, China

**Keywords:** *Bombyx mori*, *BmEcKL1*, CRISPR/Cas9, middle silk gland, posterior silk gland

## Abstract

The silkworm (*Bombyx mori*) has served humankind through silk protein production. However, traditional sericulture and the silk industry have encountered considerable bottlenecks and must rely on major technological breakthroughs to keep up with the current rapid developments. The adoption of gene editing technology has nevertheless brought new hope to traditional sericulture and the silk industry. The long period and low efficiency of traditional genetic breeding methods to obtain high silk-yielding silkworm strains have hindered the development of the sericulture industry; the use of gene editing technology to specifically control the expression of genes related to silk gland development or silk protein synthesis is beneficial for obtaining silkworm strains with excellent traits. In this study, *BmEcKL1* was specifically knocked out in the middle (MSGs) and posterior (PSGs) silk glands using CRISPR/Cas9 technology, and Δ*BmEcKL1*-MSG and Δ*BmEcKL1*-PSG strains with improved MSGs and PSGs and increased silk production were obtained. This work identifies and proves that *BmEcKL1* directly or indirectly participates in silk gland development and silk protein synthesis, providing new perspectives for investigating silk gland development and silk protein synthesis mechanisms in silkworms, which is of great significance for selecting and breeding high silk-yielding silkworm varieties.

## 1. Introduction

With the publication of the silkworm (*Bombyx mori*) genome sketch in 2004 [1], a new era of silkworm research began, followed by a series of innovative research efforts that yielded genome fine maps [2], genetic variation maps [3], gene chips [4], and epigenetic maps [5], elevating silkworm research. In this context, transgenic silkworm technologies have been gradually established and have contributed immensely to the study of silkworm functional genomics [6,7,8]. CRISPR/Cas9 gene editing technology has become one of the important tools for studying silkworm functional genomics and utilizing it to improve and cultivate new high-quality silkworm strains is paramount.

The economic value of silkworms is primarily reflected in the application of silk proteins. The silk gland is the only organ in the silkworm capable of secreting silk proteins and is divided into the anterior (ASGs), middle (MSGs), and posterior (PSGs) silk glands according to their function and structure. The MSG mainly secretes sericin, which comprises sericin 1 (Ser1), 2 (Ser2), and 3 (Ser3) proteins according to their molecular weights [9]. The PSG is capable of secreting silk fibroin proteins, which mainly include silk fibroin heavy chain protein (Fib-H), silk fibroin light chain protein (Fib-L), and P25 protein [10,11,12]. The silk fibroin proteins secreted by the PSG are enveloped in the silk sericin protein secreted by the MSG, compressed by the ASG, and ejected from the body to form silk. The development of silk glands in the silkworm can be roughly divided into three stages: silk gland development in the embryonic and larval stages and silk gland degradation in the pupal stage [13,14,15]. In the embryonic stage, silk gland cells undergo approximately 10 mitotic divisions and differentiation of the MSG and PSG to form fully developed silk gland tissue approximately 8 days after egg laying [16]. Cyclins B and E and Scr have been shown to be involved in this process [17,18]. In the larval stage, the silk gland no longer undergoes mitosis and increases its DNA content only through intranuclear replication [19,20]; it has been shown that Let7 MicroRNAs (Let7) knockout promotes intranuclear replication in silk gland cells [21,22]. Apoptosis in the silk gland is thought to be regulated by ecdysteroids (insect growth and molting hormones) and involves both apoptosis and autophagy [23,24,25]. Silk gland development and silk protein synthesis require the involvement of a large number of genes. Although several studies have been conducted, the mechanisms by which the relevant genes regulate silk gland development or affect silk protein synthesis remain unexplored. Studying silk gland development and silk protein synthesis mechanisms will help us better understand the relationship between silk gland development and silk yield, provide theoretical guidance for the genetic improvement of high silk-yielding silkworm varieties, and facilitate the research and development of new materials.

Ecdysteroid kinase-like-1 (*EcKL1*) is a member of the ecdysteroid kinase-like family (*EcKLs*), which contains a Zinc finger C4 and HLH domain containing the kinase domain subfamily of choline kinases (CHK kinase-like) with a structural domain similar to ecdysteroid 22 kinase (EcKinase). Most insects contain 12–105 genes encoding this family. This family is believed to include kinases that act on other small molecule substrates and may function in detoxification processes [26]. Mutations in five *EcKLs* in the Drosophila One ancestral *EcKL* clade in the Drosophila (*Dro5*) branch significantly reduce developmental tolerance to caffeine and the fungal secondary metabolite tretinoin in Drosophila [27]. Overexpression of Juvenile hormone-inducible protein 26 (*JHI-26*) in the testes of male flies leads to paternal-associated lethality and reduces mating acceptability by females with whom they mate [28,29]. *EcKLs* genes do not seem to have received much attention in the silkworm, except for the identification of *EcKinase*, an enzyme responsible for phosphorylating ecdysteroids at C-22 in the ovary to form the physiologically inactive ecdysteroid 22-phosphates [30,31,32]. Other *EcKL* genes have remained uncharacterized until recently when researchers discovered that the silk gland of the silkworm is affected by Let-7, a gene that affects silk gland development. Let-7 can negatively regulate EcKinase by binding to it [21], suggesting that other EcKLs possessing structural domains similar to those of EcKinase have potential regulatory roles in the growth and development of silkworm silk glands.

This study aimed to determine the role of *BmEcKL1* in silk gland development and silk protein synthesis in silkworms. We screened the potential target gene *BmEcKL1* affecting silk gland development by analyzing the spatiotemporal expression of 14 *EcKLs* genes in the silkworm. We successfully knocked out *BmEcKL1* in the MSG and PSG using CRISPR/Cas9 gene editing technology, demonstrating that *BmEcKL1* is involved in regulating the growth and development of silkworm silk glands, and simultaneously affects silk protein synthesis to a certain extent.

## 2. Results

### 2.1. Expression Analysis of EcKL Genes in the Silkworm

**Figure 1:**
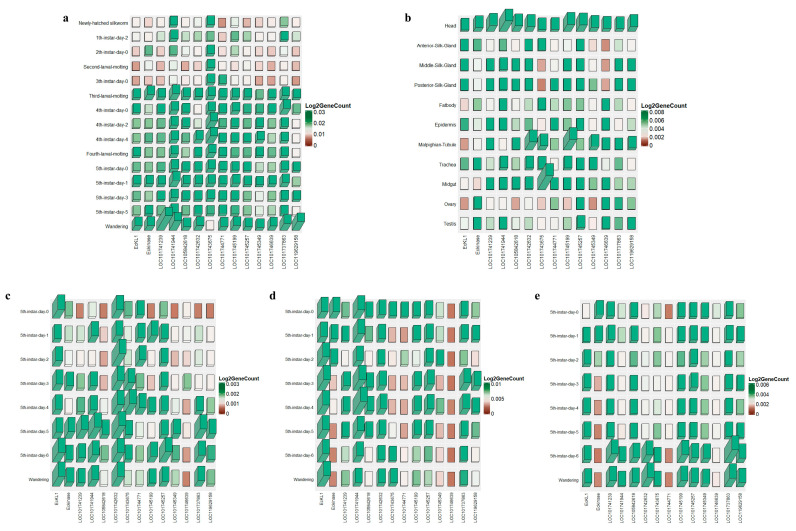
Spatiotemporal expression of *EcKLs*. *EcKL1* exhibited superior expression in the ASG, MSG, and PSG. (**a**) Period expression profile of *EcKLs* during the entire larval stage. (**b**) Tissue expression of *EcKLs* in the day 3 fifth instar. (**c**) Fifth-instar temporal expression of *EcKLs* in the anterior silk gland. (**d**) Fifth-instar temporal expression of *EcKLs* in the middle silk gland. (**e**) Fifth-instar temporal expression of *EcKLs* in the posterior silk gland.

*EcKL* genes seem to be rich in functionality, but very few studies focus on the silkworm. Based on the known information, it is difficult to select suitable research genes from numerous genes. Therefore, it is necessary to analyze the spatiotemporal expression of the *EcKL* family to help us select the genes for study. The expression profile during the entire silkworm period (Figure 1a) showed that most *EcKL* genes had low expression from the newly-hatched silkworm to the third instar, and expression suddenly increased from the third instar of sleeping silkworms until it reached a maximum during the wandering period. The expression profile of the 5th-instar day 3 tissues showed that the *EcKL* genes were highly expressed primarily in the head, and most were expressed in the malpighian-tubule, midgut, MSG, and PSG (Figure 1b). LOC10174263, LOC101743675, LOC101745199, and LOC101745257 were highly expressed in either the midgut or the malpighian-tubule, implying that they could be related to detoxification. In contrast, *EcKL1* and *EcKinase* expression was lower in both tissues, but *EcKL1* exhibited superior expression in the ASG, MSG, and PSG. We mapped the expression profiles of the *EcKLs* in the ASG, MSG, and PSG at the fifth instar and revealed that *EcKL1*, *LOC101742632*, and *LOC101745257* were all expressed in the ASG (Figure 1c). In addition, the MSG full-period expression profile (Figure 1d) demonstrated high expression of *EcKL1* and *LOC101741944*, while that of the PSG (Figure 1e) exhibited a more homogeneous expression pattern. Therefore, according to these results, we selected the *EcKL1* gene, which had a good expression profile in the silk gland, as the target of the next study.

### 2.2. BmEcKL1 Knockout in MSG and PSG

**Figure 2:**
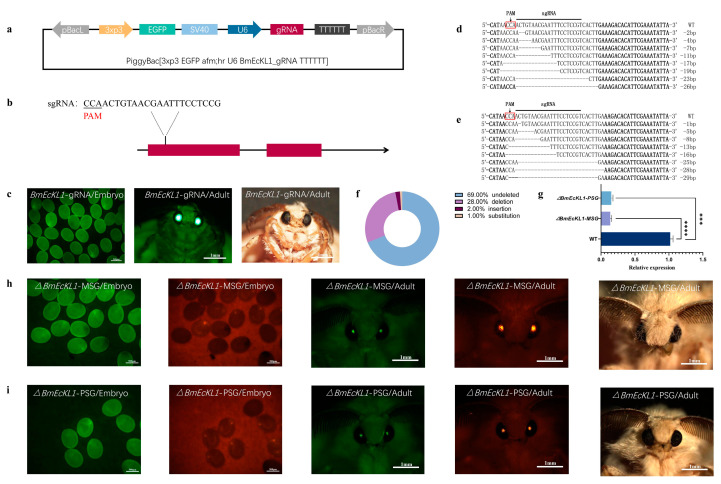
Construction of sgRNA strains and mutants. Relative expression means the expression of the experimental gene relative to the reference gene sw22934. (**a**) Schematic diagram of the sgRNA transgenic vector. (**b**) The designed sgRNA is located in the first exon. (**c**) Positive individuals selected at the G1 embryo and adult stages by screening for the EGFP marker that emit green light in the eyes. (**d**) Sequence alignment of sgRNA-targeted genomic regions in the Δ*BmEcKL1*-MSG strain exhibits multiple forms of mutations. (**e**) Sequence alignment of sgRNA-targeted genomic regions in the Δ*BmEcKL1*-PSG strain exhibits multiple forms of mutations. (**f**) Ratios of different mutation types. (**g**) Expression of *BmEcKL1* at the transcriptional level. (**h**) Positive individuals of the Δ*BmEcKL1*-MSG strain were selected at the F1 embryo and adult stages by screening for green-emitting EGFP and red-emitting RFP markers in the eyes. (**i**) Positive individuals of the Δ*BmEcKL1*-PSG strain were selected at the F1 embryo and adult stages by screening for green-emitting EGFP and red-emitting RFP markers in the eyes. Significant differences were defined as *** *p* < 0.001, **** *p* < 0.0001.

The *BmEcKL1* gene has a full CDS length of 1230 bp and contains two exons. Figure 2a shows the knockout vector structure diagram. The constructed vector was injected into silkworm eggs within 2 h after the eggs were laid, and the injected eggs were referred to as the G0 generation. The G0-generation silkworms were reared to adulthood and mated with wild-type silkworms (WT), and the laid eggs were referred to as the G1 generation. The eggs were identified by detecting green fluorescent protein (EGFP) expression in the eyes under the control of neuron-specific 3 × P3 promoter on the sixth day after the eggs of the G1 generation were laid. The G1 generation positive for green fluorescent eyes was reared to adulthood and rescreened (Figure 2c), and a positive gRNA strain with green fluorescent eyes was obtained and designated *BmEcKL1*-gRNA.

The *BmEcKL1*-gRNA G1 positive strains were then crossed with PSG-Cas9 and MSG-Cas9 [33,34] strains to produce F1 generations, which were screened for green and red fluorescent markers in the eyes of late-stage embryos and adults (Figure 2h,i); the positive F1 generations obtained were designated Δ*BmEcKL1*-PSG and Δ*BmEcKL1*-MSG, respectively. To verify whether mutation occurred in the *BmEcKL1* gene sequence, the fragment containing the sgRNA target site was PCR amplified with site-specific primers. The results showed that various forms of DNA fragment deletions occurred at the sgRNA loci in Δ*BmEcKL1*-MSG- and Δ*BmEcKL1*-PSG-positive individuals (Figure 2d,e). Subsequently, we conducted experiments using mutants with deleted bases greater than 15 and not multiples of 3. Further analysis of the sequencing results showed that the individual knockout efficiency was 31% (Figure 2f), of which 28% were fragment deletions, 2% were fragment insertions, and 1% were base substitutions. Finally, the expression of *BmEcKL1* at the transcriptional level was measured in the WT, Δ*BmEcKL1*-MSG, and Δ*BmEcKL1*-PSG strains using *EcKL1* mutant primers (Figure 2g), which showed that the *BmEcKL1* was successfully knocked out in both Δ*BmEcKL1*-MSG and Δ*BmEcKL1*-PSG individuals.

### 2.3. Deletion of BmEcKL1 Leads to MSG Abnormalities and Changes in Silk Protein Production

**Figure 3:**
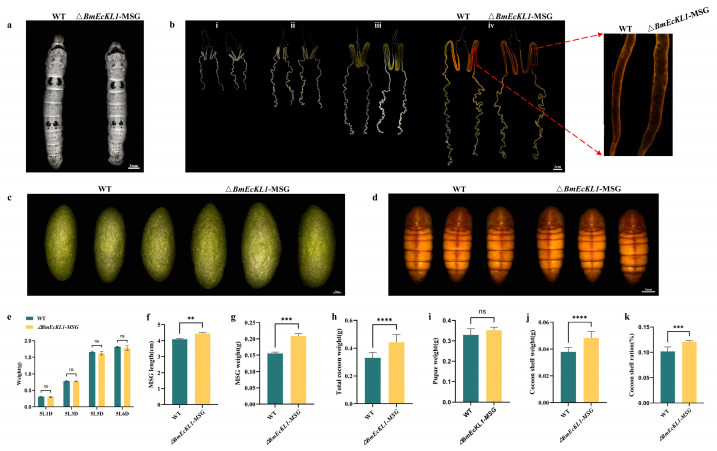
Phenotypic statistics of the Δ*BmEcKL1*-MSG strain. For each set of experiments, 50 WT and 50 mutant silkworms were counted and performed three times in total. (**a**) Individual size differences between the WT and Δ*BmEcKL1*-MSG; there is no significant difference. (**b**) Silk gland phenotype of the Δ*BmEcKL1*-MSG at the fifth instar ((**i**) 5L1D; (**ii**) 5L3D; (**iii**) 5L5D; (**iv**) 5L6D); the mutant has a larger MSG. (**c**) The cocoon phenotype is slightly larger in the mutant pupae than in the WT. (**d**) The pupae phenotype; there is no significant difference. (**e**) WT and Δ*BmEcKL1*-MSG strains were weighed at the fifth-instar larva stage; there is no significant difference. (**f**) MSG length of the Δ*BmEcKL1*-MSG strain. (**g**) MSG weight of the Δ*BmEcKL1*-MSG strain. (**h**) Total cocoon weight of the Δ*BmEcKL1*-MSG strain. (**i**) Pupae weight of the Δ*BmEcKL1*-MSG strain. (**j**) Cocoon shell weight of the Δ*BmEcKL1*-MSG strain. (**k**) Cocoon shell ratio of the *ΔBmEcKL1*-MSG strain. Significant differences were defined as ** *p* < 0.01, *** *p* < 0.001, **** *p* < 0.0001. The meaning of ns is that there is no significant difference.

WT and Δ*BmEcKL1*-MSG individuals were mixed and reared to the fifth instar, and no significant differences in the changes in individual size and body weight were observed on day 3 of the fifth instar (5L3D; Figure 3a,e). Next, the larvae were dissected at different time points at the fifth instar stage, and the silk glands were assessed and visualized using a camera. At day 1 of the fifth instar (5L1D), the MSG size of the Δ*BmEcKL1*-MSG strain was almost the same as that of the WT (Figure 3b(i)) but slightly larger than that of the WT at 5L3D (Figure 3b(ii)). By 5L5D, the MSGs of the Δ*BmEcKL1*-MSG and WT clearly differed, with longer and larger Δ*BmEcKL1*-MSG M-MSGs and P-MSGs (Figure 3b(iii)). Compared with the WT, the Δ*BmEcKL1*-MSG P-MSG was significantly thicker and appeared roughly serrated on the surface by 5L6D (Figure 3b(iv)).

For a more comprehensive assessment, we measured the MSG length and weight at 5L6D. The MSG of the Δ*BmEcKL1*-MSG strain increased by approximately 10% (Figure 3f), while its weight increased by approximately 30% relative to those of the WT (Figure 3g). To understand whether the changes in the silk glands affect silk protein secretion, we determined cocoon and pupa phenotypes after clustering, and the results showed that the cocoons of the Δ*BmEcKL1*-MSG strain were slightly larger than those of the WT (Figure 3c). In addition, the full cocoon weight and layer weight increased to some extent (Figure 3h,j), but the difference in pupal size and weight between the two strains did not significantly differ (Figure 3d,i). Therefore, the cocoon layer ratio in the Δ*BmEcKL1*-MSG strain marginally increased (Figure 3k).

### 2.4. BmEcKL1 Knockout Leads to Enlarged PSG and Increased Silk Yield

**Figure 4:**
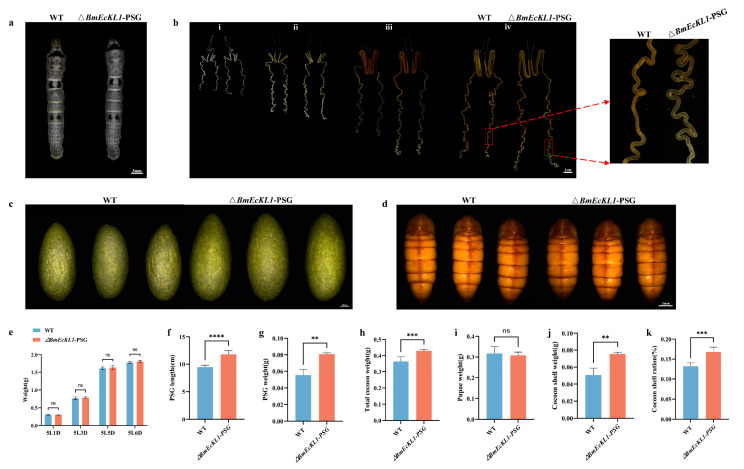
Phenotypic statistics of the *ΔBmEcKL1*-PSG strain. For each set of experiments, 50 WT and 50 mutant silkworms were counted and performed three times in total. (**a**) Individual size difference between the WT and Δ*BmEcKL1*-PSG; there is no significant difference. (**b**) Silk gland phenotype of Δ*BmEcKL1*-PSG at the fifth-instar larva stage ((**i**) 5L1D; (**ii**) 5L3D; (**iii**) 5L5D; (**iv**) 5L6D); the mutant has a longer PSG. (**c**) The cocoon phenotype is significantly larger in the mutant pupae than in the WT. (**d**) The pupae phenotype; there is no significant difference. (**e**) WT and Δ*BmEcKL1*-PSG strains were weighed at the fifth-instar larva stage; there is no significant difference. (**f**) PSG length of the Δ*BmEcKL1*-PSG strain. (**g**) PSG weight of the Δ*BmEcKL1*-PSG strain. (**h**) Total cocoon weight of the Δ*BmEcKL1*-PSG strain. (**i**) Pupa weight of the Δ*BmEcKL1*-PSG strain. (**j**) Cocoon shell weight of the Δ*BmEcKL1*-PSG strain. (**k**) The cocoon shell ratio of the Δ*BmEcKL1*-PSG strain. Significant differences were defined as ** *p* < 0.01, *** *p* < 0.001, **** *p* < 0.0001. The meaning of ns is that there is no significant difference.

WT and Δ*BmEcKL1*-PSG were mixed and reared to the fifth instar. We recorded the changes in individual size and body weight of the WT and Δ*BmEcKL1*-PSG strain at 5L3D, and the results showed that none of them appeared to be significantly altered (Figure 4a,e). To determine whether *BmEcKL1* knockout affects PSG development, we dissected and visualized the silk glands at the fifth instar. The results showed that at 5L1D, the PSG of the Δ*BmEcKL1*-PSG strain had changed minimally compared with that of the WT (Figure 4b(i)), and from 5L3D onwards, the change was more apparent. The PSG of the Δ*BmEcKL1*-PSG strain was longer than that of the WT (Figure 4b(ii)), and by 5L5D, it had become longer and curlier (Figure 4b(iii)), becoming significantly longer and curlier by 5L6D (Figure 4b(iv)). Moreover, we measured the PSG length and weight at 5L6D of the Δ*BmEcKL1*-PSG strain. We observed that the former increased by approximately 20% (Figure 4f) and the latter by approximately 38% (Figure 4g) relative to those of the WT. Furthermore, we assessed the cocoon and pupa phenotypes and found that the Δ*BmEcKL1*-PSG cocoons were larger than those of the WT (Figure 4c), with an increase in full cocoon weight of approximately 10% (Figure 4h). The cocoon layer weight was also significantly increased (Figure 4j); nevertheless, pupae did not significantly differ (Figure 4d,i). Thus, the cocoon layer ratio of the Δ*BmEcKL1*-PSG strain was significantly increased (Figure 4k).

### 2.5. BmEcKL1 Knockout Alters Silk Protein Secretion

**Figure 5:**
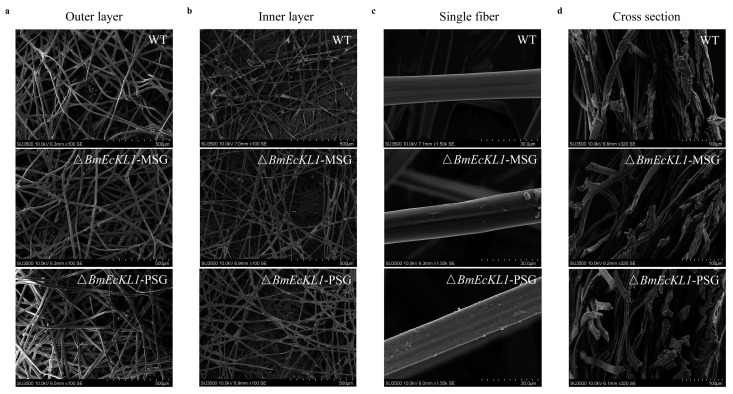
Scanning electron microscopy images of the WT, Δ*BmEcKL1*-MSG, and Δ*BmEcKL1*-PSG cocoons. (**a**) The first column shows the outer surface; the mutant outer surface is denser than the WT. (**b**) The second column shows the inner surface; the mutant inner surface is denser than the WT. (**c**) The third column shows a single fiber; the mutant single fiber is coarser than the WT. (**d**) The fourth column shows the cross-section, the mutant has a thicker cross-section than the WT.

To investigate whether *BmEcKL1* knockout affects the secreted silk protein, we analyzed the cocoons of the WT, Δ*BmEcKL1*-MSG, and Δ*BmEcKL1*-PSG strains by scanning electron microscopy (SEM). The results showed that the silk fibers of the Δ*BmEcKL1*-MSG and Δ*BmEcKL1*-PSG strains were thicker than those of the WT. The outer and inner layers of both the Δ*BmEcKL1*-MSG and Δ*BmEcKL1*-PSG cocoons were denser than those of the WT (Figure 5a,b), whereas those of the Δ*BmEcKL1*-PSG strain were less dense relative to those of the Δ*BmEcKL1*-MSG strain. This was corroborated by the observation of single filament fibers (Figure 5c).

**Figure 6:**
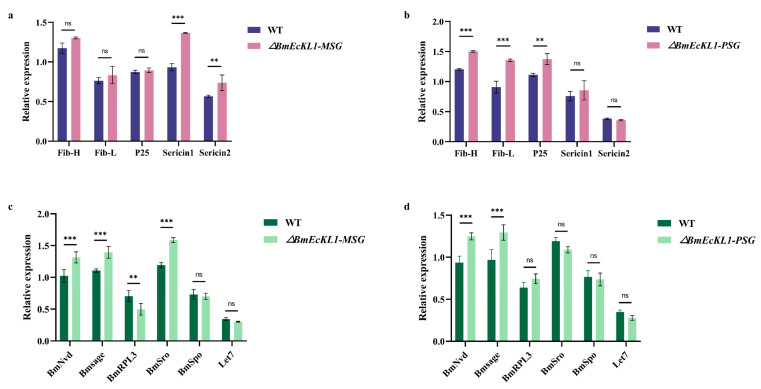
Transcriptional level analysis of related genes. For each set of experiments, 5 WT and 5 mutant silkworms were counted and performed three times in total. Relative expression means the expression of the experimental gene relative to the reference gene sw22934. (**a**) The transcription levels of major silk protein genes in the Δ*BmEcKL1*-MSG strain. (**b**) The transcription levels of major silk protein genes in the Δ*BmEcKL1*-PSG strain. (**c**) The transcription levels of genes involved in silk gland development in the Δ*BmEcKL1*-MSG strain. (**d**) The transcription levels of genes involved in silk gland development in the Δ*BmEcKL1*-PSG strain. Significant differences were defined as ** *p* < 0.01, *** *p* < 0.001. The meaning of ns is that there is no significant difference.

The single filament fibers of the Δ*BmEcKL1*-MSG and Δ*BmEcKL1*-PSG strains were coarser than those of the WT, while those of the Δ*BmEcKL1*-PSG strain were coarser than those of the Δ*BmEcKL1*-MSG strain. In addition, the cross-sections of the Δ*BmEcKL1*-MSG and Δ*BmEcKL1*-PSG cocoons were thicker than those of the WT cocoons (Figure 5d). *BmEcKL1* knockout appeared to enhance the performance of silk. To further investigate the reasons behind this phenomenon, we determined the expression of Fib-H, Fib-L, P25, sericin-1, and sericin-2, the main silk proteins secreted by silk glands, at the transcriptional level at 5L6D. The results showed no significant change in fibroin in the Δ*BmEcKL1*-MSG strain, whereas sericin-1 and sericin-2 were significantly increased (Figure 6a). In the Δ*BmEcKL1*-PSG strain, three fibroin proteins, Fib-H, Fib-L, and P25, were upregulated to varying degrees. However, the change in sericin was not significant (Figure 6b). Finally, we determined the transcriptional expression of genes related to silk gland development and hormone metabolism. The results showed that *BmNvd*, *Bmsage*, and *BmSro* expression increased in the Δ*BmEcKL1*-MSG strain, whereas *BmRPL3* expression was downregulated, and *BmSpo* and *Let7* did not significantly change. *BmNvd* and *Bmsage* expression was upregulated in the Δ*BmEcKL1*-PSG strain, whereas that of the others did not change significantly.

## 3. Discussion

In this study, we demonstrated that *BmEcKL1* knockout affects silk gland development as well as silk protein synthesis. Using CRISPR/Cas9 technology, *BmEcKL1* was knocked out in the MSG and PSG. On the one hand, *BmEcKL1* knockout resulted in an enlarged and less smooth MSG and increased sericin protein production. However, although the cocoon size increased, the cocoon layer ratio only increased slightly. On the other hand, *BmEcKL1* knockout resulted in an elongated and curlier PSG and higher fibroin protein production. Furthermore, the cocoon layer ratio considerably improved with the increase in cocoon size.

The silkworm is a valuable economic insect and model lepidopteran, occupying an important position in human history, culture, and economic life. Silkworms are characterized by silk ejection and cocooning, and the growth and development of the silk gland, the only silk-producing organ of silkworms, is closely related to silk protein synthesis. Silk glands are larger in high silk-producing strains compared with low silk-producing strains [35]. Transgenic techniques have revealed that Ras1 or Yorkie overexpression can increase silk gland size and improve silk production [36,37]. However, silk gland development is an extremely complex and synergistic process that involves hormone regulation, energy metabolism, material conversion, and other important processes requiring the participation of a large number of genes. The successive expression of genes, such as cyclins B and E and SGF1, regulates the formation and extension of the silk gland in the embryonic stage. The development of the silk gland in the larval stage is primarily affected by nuclear replication and silk gland cell size. Let7 knockout promotes the nuclear replication of silk gland cells, leading to silk gland enlargement and increased silk production. Collectively, both silk gland cell volume expansion and the intranuclear replication of DNA ultimately prepare the organ for the synthesis of large amounts of silk proteins at the fifth instar. In the present study, we knocked out *BmEcKL1* in the MSG and PSG using CRISPR/Cas9 technology, which led to their expansion and improved silk production. Nevertheless, the Δ*BmEcKL1* MSG strain was inferior to the Δ*BmEcKL1* PSG strain in terms of the increase in silk yield. The difference between the two strains may be attributed to the fact that fibroin proteins account for a greater proportion of silk fibers than do sericin proteins, and the increase in fibroin can improve the cocoon layer ratio more under the same conditions.

The EcKLs have a highly conserved cluster, HxDhx3Nh3… D, known as the Brenner motif (where h denotes a large hydrophobic amino acid and x denotes any amino acid) [38,39], that is an important structure for this family of proteins to function, shared by several protein kinases [40], choline kinases [41,42], and aminoglycoside phosphotransferases [43]. EcKinase, as a representative of the EcKLs, has previously been shown to bind directly to ecdysone. Before thymus differentiation, free ecdysteroids are involved in controlling early events in embryonic development, including embryonic epidermogenesis and germ band lengthening, and are thought to be released from ovarian-derived ecdysteroid phosphates [44]. Considering how ecdysteroid phosphate is produced, researchers have suggested that in mature oocytes, EcKinase, which is responsible for the phosphorylation of ecdysteroid at the C-22 position, converts ecdysteroids into inactive phosphate. The larval *EcKL* expression profiles throughout the period exhibited some regularity. The analysis of *BmEcKL1* expression showed that starting from the third instar, *BmEcKL1* expression increased at the late stage of each instar, both at the early stage of molting and at the pupal transformation stage, which seemed to be similar to the periodic change of the 20-hydroxyecdysone (20E) titer. The transcript levels of silk gland development-related genes and 20E synthase were examined in the Δ*BmEcKL*1-MSG and Δ*BmEcKL1*-PSG strains. The results showed that 20E synthesis-related genes were elevated in both knockout lines, implying that *BmEcKL1* might be directly or indirectly regulated by 20E, in addition to *Bmsage*, which is thought to control the number of silk gland cells through the cell cycle pathway. The expression of this gene in the knockout lines appeared to be altered by *BmEcKL1* knockout; therefore, we hypothesized that *BmEcKL1* knockout altered the energy metabolism pathway of the relevant hormones in the silkworm, causing changes in the relevant genes regulating silk gland development, such as *Bmsage* [45].

Silk is a natural animal protein, mainly comprising two types of proteins: fibroin and sericin. On the one hand, fibroin is a high molecular weight insoluble fibrous protein, and the current silk industry mainly utilizes it to produce raw silk and silk products. On the other hand, sericin is a low molecular weight soluble globular protein that has moisturizing, antioxidant, anti-bacterial, and UV-resistant functions, among others, and has high development and utilization value in the fields of cosmetics, medicine, food, and fiber-modification materials [46,47]. Fibroin and sericin account for 70–80% and 20–30%, respectively, in conventional varieties [48]. The cocoons of both Δ*BmEcKL1*-MSG and Δ*BmEcKL1*-PSG strains were enlarged to a certain extent, and SEM revealed that the silk fibers of both strains were coarser than those of the WT, with those of the Δ*BmEcKL1*-PSG strain being the coarsest. Thus, the Δ*BmEcKL1*-PSG strain silk fibers have superior toughness, whereas those of the Δ*BmEcKL1*-MSG strain have wider utility in more fields.

In conclusion, silk yield is paramount to the silk industry. Previously, high silk-yielding silkworm strains were obtained through traditional genetic breeding methods, which are labor- and time-intensive. However, the development and application of gene editing technology [49,50,51] have enabled researchers to produce silkworms with superior economic traits more conveniently, quickly, and efficiently by regulating silk gland development and silk protein synthesis. In this study, we demonstrated, that *BmEcKL* genes are involved in the regulation of silk gland development and silk protein synthesis, providing a new direction for future research on *EcKLs* in insects and a theoretical basis for the mechanism of silk gland development and silk protein synthesis mechanisms. Furthermore, our findings provide a reference for the selection and breeding of high silk-yielding silkworms. However, further studies are needed to fully elucidate how *BmEcKL1* is involved in regulating silk gland development and silk protein synthesis. Meanwhile, whether knockout efficiency can be improved by designing multiple SgRNA tandem vectors, which will lead to greater changes in the silk glands and higher silk production, remains to be verified. Finally, whether other *EcKLs* genes also regulate silk gland development needs to be further investigated.

## 4. Materials and Methods

### 4.1. Bioinformatics Analysis

The full-length cDNA and protein sequences (ACCESSION: XM_012688665, XP_012544119) of *BmEcKL1* were obtained from the NCBI database. *BmEcKL1* was characterized and sequenced using ExPASy. Available online: http://www.expasy.org/ (accessed on 10 February 2023) [52], and Interpro. Available online: http://www.ebi.ac.uk/interpro/ (accessed on 15 February 2023) [53]; the protein structural domains were analyzed using SMART. Available online: http://smart.embl-heidelberg.de/ (accessed on 21 February 2023) [54]; multiple sequence comparison analysis was conducted using Clustal 2.1.

### 4.2. Silkworm Rearing

The multivoltine, non-lagging silkworm strain D9L was used in this study. The silkworms were reared at the Biological Research Center, Institute of Frontier and Interdisciplinary Sciences, Southwest University of China. The rearing of silkworms was divided into 10 zones, with 150 silkworms per zone. Silkworm larvae were reared on fresh mulberry leaves (*Morus* spp.) under photoperiod conditions of 12 h light/12 h dark at 25 ± 1 °C and 70 ± 5% humidity.

### 4.3. RNA Extraction and RT-qPCR Validation

Silkworms from a certain zone were randomly selected for sampling, and 5 silkworms from each period and tissue were selected and mixed as one sample. Sample RNA was extracted using TRIzol™ reagent (Invitrogen, Carlsbad, CA, USA), and reverse transcription was performed using a PrimeScript™ RT reagent Kit with gDNA Eraser (Takara Bio Inc., Shiga, Japan). All samples were stored at −80 °C. Three independent replicates were performed for each RNA extraction. Primers were designed using Primer Premier 5.0; all designed primers are listed in Supplementary Table S1. Quantitative PCR (qPCR) was performed using SYBR^®^Premix Ex Taq™II (Takara Bio Inc., Shiga, Japan) under the following conditions: 95 °C for 10 s, followed by 40 cycles of 95 °C for 5 s, and 60 °C for 30 s. *B. mori sw22934* [55] was used as an internal reference gene. Three independent replicates were performed for each RT-qPCR experiment.

### 4.4. Vector Construction and Microinjection

The *BmEcKL1* exon sequence was selected as the target region for knockout. SgRNA was designed using CCtop (available online: https://www.cos.uni-heidelberg.de/en (accessed on 16 May 2022) [56]) on the first exon (Figure 2b) and used for sequence synthesis, after which the synthesized sequence was annealed to form double-stranded SgRNA. Restriction endonuclease AarI was used to cut the piggyBac (3 × P3-EGFP-SV40-U6-TTTTTT) base vector and the double-stranded gRNA was connected to the piggyBac (3 × P3-EGFP-SV40-U6-TTTTTT) base vector after enzyme digestion [57] to construct a piggyBac (3 × P3-EGFP-SV40-U6-gRNA-TTTTTT) knockout vector (the base vector was provided by the Biological Research Center, Institute of Frontier and Interdisciplinary Sciences, Southwest University of China). The knockout vector plasmid and helper plasmid were mixed at a molar ratio of 1:1 at a concentration of 400 ng/uL, and injected into silkworm eggs with an injection volume of 0.1–0.2 uL per egg. Finally, the injected eggs were stored at 25 ± 1 °C and 70 ± 5% humidity.

### 4.5. Phenotype Statistics and Observation

The transgenic silkworms were screened using a fluorescence microscope (Olympus, Tokyo, Japan), and the genome was extracted as a template. PCR amplification was performed with *BmEcKL1* target site detection primers (Table S1). The PCR fragment was subjected to agarose gel electrophoresis, purified, connected to T vector, transformed in *E. coli*, and single clones were sequenced by Sanger method using M13F/R primer. WT and transgenic silkworms were reared to the fifth instar for dissection, after which the silk glands were excised, washed with phosphate-buffered saline, and imaged using a fluorescence microscope (Olympus, Tokyo, Japan). The WT and transgenic silkworms were mixed and reared to the fifth instar, and their body weights were counted. The silkworms were reared to the moth stage, and cocoons were cut in advance to identify males and females.

### 4.6. Scanning Electron Microscopy

Cocoon morphology was assessed using a scanning electron microscope (JSM-5000; JEOL, Tokyo, Japan). The cocoon samples of each group were glued to conductive carbon tape and coated with a thin layer of platinum (NeoCoater MP-19020NCTR; JEOL). SEM observations were performed at 25 ± 1 °C.

### 4.7. Statistics and Reproducibility

All statistical analyses were performed using GraphPad Prism 8.0 (GraphPad Software, La Jolla, CA, USA), and the data are presented as the means ± SEM. The mean cycle threshold (Ct) value was converted to the relative expression level using the 2^−ΔΔCt^ method [58,59]. Statistical analyses of the expression levels were performed using a two-tailed unpaired student *t*-test. Significant differences were defined as * *p* < 0.05, ** *p* < 0.01, *** *p* < 0.001, **** *p* < 0.0001.

## Data Availability

All of the data used in this study have been provided in the main text and the Supplementary Materials.

## Supplementary Materials

The following supporting information can be downloaded at: https://www.mdpi.com/article/10.3390/ijms25031907/s1.
