# Supplementary material for: CRISPR/Cas9-Mediated Editing of BmEcKL1 Gene Sequence Affected Silk Gland Development of Silkworms (Bombyx mori)
Source: Int J Mol Sci. 2024 Feb 5;25(3):1907. doi: 10.3390/ijms25031907 (PMC10856159; doi:10.3390/ijms25031907)
Supplement: Supplementary file 1 [file ijms-25-01907-s001.zip › ijms-2813123-supplementary.pdf]

Table S1. Table S1. Presents all the primers used in this article.

Name	sequence
EcKL1 RT-qPCR primers	F:TGCACAAATGGTGGAAACTATC<br>R:TCTTTCATCGGCAGGTACTTTA
Eckinase RT-qPCR primers	F: CCTCCACTACGGGTGTCCTAT<br>R: TTTTCTGAACGCCTGGTCC
LOC101742632 RT-qPCR primers	F: CCTCCACTACGGGTGTCCTAT<br>R: TTTTCTGAACGCCTGGTCC
LOC101743675 RT-qPCR primers	F:CGCCCCTCAAAATGAAAAC<br>R:CGGCAAATCTGAATCTGTCG
LOC105842618 RT-qPCR primers	F: CTCGACTCCAAGCCTAAACATG<br>R: CGAAGGACAAAGCGTGAAACT
LOC101745257 RT-qPCR primers	F: AATGCCTGCCTGATAGATTACC<br>R: GTCGGAGTTCAGTGTCAGTGCTA
LOC101744771 RT-qPCR primers	F: GAAGCACGTCCAGTGCGTAT<br>R: TTTGAGGCTCGGAACTTTGA
LOC101746639 RT-qPCR primers	F: ACGCATTATCTTTCGTCTGGG<br>R: TGATTAGGGACGCGGCTTA
LOC101745349 RT-qPCR primers	F: GAACGTAGTGGCACATCGTGA<br>R: ACAAGTCCAGCATCGGAGAAC
LOC101741239 RT-qPCR primers	F: AATCGGCTACAACTGGGAGG<br>R: TGCAGCAGTCTTCGGATCTAAT
LOC101737863 RT-qPCR primers	F: AACTGCGTTTTGCTGAAGGA<br>R: TGGAAGTGCGCTAAATGTCTC
LOC101745199 RT-qPCR primers	F: CGTTGGAGTCAGGTTTGGC<br>R: TGGATTACCAGTTCTGTCTTCGT
LOC119629158 RT-qPCR primers	F: TTATGGTTGTTATCTGGACGGC<br>R: TTCGCACTTTGTAGGGTGTTTC
LOC101741944 RT-qPCR primers	F: CTTGCCCACGCTGACTTTT<br>R: TCGTCCATTGGCTTTCCTAA
FibH RT-qPCR primers	F: TCTGTGTCATCTGCTTCATCTCG<br>R: TATCCAGGACGAAGTAAGAAAC
FibL RT-qPCR primers	F:GGCAGATAGATGGGCGATAAT<br>R:GTCGATGACACTGACAAAAGCA
P25 RT-qPCR primers	F:CGTAGGTGGCGTTGAAGTATG<br>R:GCCGCGATCTCGAAATGTA
Sericin1 RT-qPCR primers	F: CACAACCGATAAGACGAG<br>R: GACGAAGTGGAGGAAGC
Sericin2 RT-qPCR primers	F: CATCGGCTGACTACCA<br>R: AGAGTTGCTGCCCTTAC
Bmsage RT-qPCR primers	F:CTTCCTTCGGTTTGTCGTCC<br>R:GGCCCCTTTGCCTCTGATA
BmRPL3 RT-qPCR primers	F:TGGCACACAAAGAAGCTACCC<br>R:TGACCAGCACGAGCTACAGTG

BmNvd RT-qPCR primers	F:TGCCTCGATACCCGAAGTC
	R:GGTGCGTGGTGGAAACAGA
BmSro RT-qPCR primers	F:CCGTTAGCCGTCTTGTAGC
	R:CGGTTTCTATTCCTTTGTGC
BmSpo RT-qPCR primers	F:GGACATCCGATCCTTCATCT
	R:TCTTCGTGTAGCACCCTGAG
let7 RT-qPCR primers	F: GTCGGCTTGTTGAGGTAGTAGG
	R: TCCGTCAGCTCGGAAAGTTAG
sw22934	F: TTTCACGGTTGGATGGCA
	R: GAGGCAGTTTCGTACTGGCTC
Target site detection primer	F: AGCGCAGCCTTATGTTATCG
	R: AACGTCCATCCTGTGAGCAGT
SgRNA	ACTGTAACGAATTTCCTCCG
